# “Neurologist's contribution to the diagnosis of sine materia respiratory insufficiency: case report”

**DOI:** 10.1186/1471-2466-12-42

**Published:** 2012-08-08

**Authors:** Cristiano Carbonelli, Marialuisa Zedde, Alberto Cavazza, Nicola Facciolongo, Francesco Menzella, Lucia Spaggiari, Luigi Zucchi

**Affiliations:** 1 Pulmonology Unit, Department of Cardiology, Thoracic and Vascular Surgery and Critical Care Medicine, Azienda Ospedaliera ASMN, Istituto di Ricovero e Cura a Carattere Scientifico, Reggio Emilia, Italy. Viale Risorgimento 80, 42123, Reggio Emilia, Italy; 2 Neurology Unit, Department of Neuromotor Physiology, Azienda Ospedaliera ASMN, Istituto di Ricovero e Cura a Carattere Scientifico, Reggio Emilia, Italy; 3 Pathology Unit, Department of Oncology, Azienda Ospedaliera ASMN, Istituto di Ricovero e Cura a Carattere Scientifico, Reggio Emilia, Italy; 4 Radiology Unit, Department of Diagnostic Imaging, Azienda Ospedaliera ASMN, Istituto di Ricovero e Cura a Carattere Scientifico, Reggio Emilia, Italy

**Keywords:** Ultrasonography, Doppler, Transcranial"[Mesh] AND "Vascular Malformations"[Mesh] AND "Diagnosis"[Mesh] AND "dyspnea"[Mesh]

## Abstract

**Background:**

Right-to-left shunt (RLS) may be the cause of marked hypoxemia, a respiratory insufficiency which is usually difficult to diagnose by respiratory physicians as it develops in the absence of an intrinsic lung disease.

**Case presentation:**

We report a case of RLS in a patient with a hepatopulmonary syndrome caused by chronic autoimmune cholangitis. RLS was suspected clinically by physical examination and by standard CT imaging and MIP reconstruction of the pulmonary vascular bed. Repeated previous transthoracic echocardiography (TTE) studies did not reveal shunts or any cardiac defect. The final diagnosis was made by means of a minimally invasive transcranial Doppler examination with the use of saline agitated with 0.5 ml of patient’s blood as contrast solution.

**Conclusions:**

Transcranial Colour-Coded Duplex Sonography (TCCS) with saline contrast medium injection is described to have a higher sensitivity than TTE and comparable to transesophageal echocardiography (TEE) in RLS diagnosis. The collaboration of neurologists in diagnosing respiratory insufficiency is very important as the examination is simple, well tolerated in comparison with the discomfort associated with transesophageal echocardiography, and minimally invasive in comparison with angiography, which is the last diagnostic procedure in this clinical scenario. In order to confirm RLS, TCCS with blood-saline contrast medium injection should be performed for the diagnosis of chronic hypoxemia for which causes are not detected with routine clinical examinations.

## Background

Among dyspneic patients, discernment of a pattern of platypnea-orthodeoxia is key to effective evaluation. Platypnea is defined as dyspnea induced by upright posture, relieved by the recumbent position. Orthodeoxia refers to arterial desaturation resulting from assuming an upright position.

A pattern of platypnea-orthodeoxia is typical of right-to-left shunts as in patients with a patent foramen ovale or with intrapulmonary vascular dilatations and shunting from hepatopulmonary syndrome.

Hepatopulmonary syndrome is characterized by RLS and arterial deoxygenation in patients with chronic liver disease in absence of an intrinsic lung disease. Technetium-99 m macro-aggregated albumin lung perfusion, contrast echocardiography, or pulmonary angiography is required to make a definite diagnosis of RLS; only the latter can reveal the anatomic origin of the shunt if direct arteriovenous communications are present [1]. The prevalence of the syndrome among such patients ranges from 13% to 47% and the mortality associated with hepatopulmonary syndrome is high, with a median survival of 10.6 months [2].

CT angiography can show suspected intrapulmonary vascular dilatations and the hepatopulmonary syndrome with the presence of centrilobular vessel–associated micronodules connected by arcade-like dilated subpleural vascular branches [3].

As in this disease the shunt is not due to a cardiac defect, transthoracic echocardiography and transesophageal echocardiography have low sensitivity and specificity in diagnosing RLS compared to neurosonological techniques, namely transcranial Doppler (TCD) and TCCS. These techniques performed with saline contrast medium have been reported to have high sensitivity and specificity for detecting right-to-left shunts [4,5]. The modality of performing contrast TCD or TCCS is well standardized in the literature [6]. Recently, an increased sensitivity of TCD agitated blood-saline study for the optimal assessment of a suspect of RLS was reported [7,8]. The increased sensitivity of TCCS or TCD and the minimal invasiveness in comparison with angiography or with the discomfort associated with the TEE suggests that these techniques would be the method of choice to diagnose RLS in the appropriate clinical scenario.

### Case presentation

A 23-year- old Caucasian woman was admitted to the hospital because of complaint of shortness of breath on exertion with a slow and progressive onset.

Her medical history included a panhypopituitarism as a result of the surgical removal of a craniopharyngioma in paediatric age, an autoimmune cholangitis, and a long-term estroprogestinic therapy for contraceptive purposes.

On admission, a marked hypocapnic hypoxemia was found on the hemogasanalysis while breathing room air (PaCO2 24 mm, PaO2 54 mm), with an increased alveolar-arterial oxygen gradient (A-a) (46 mmHg).

The clinical evaluation of chest did not reveal any abnormality suggestive of a primary respiratory disease.

Platypnea-orthodeoxia was noted, as was worsening of the shortness of breath associated to arterial desaturation while sitting in an upright position from a recumbent position.

Spirometric assessment showed a mild restrictive pattern, with a reduced FVC (3.27 L, 77% of the predicted value) in the absence of a reduced FEV1 to FVC ratio. Desaturation was observed in a six-minute walking test. A marked reduction of DLCO (28% of predicted value) and a reduced DL to VA ratio (46% of predicted value) were suggestive of a true interstitial disease or of a pulmonary vascular disease.

**Figure 1:**
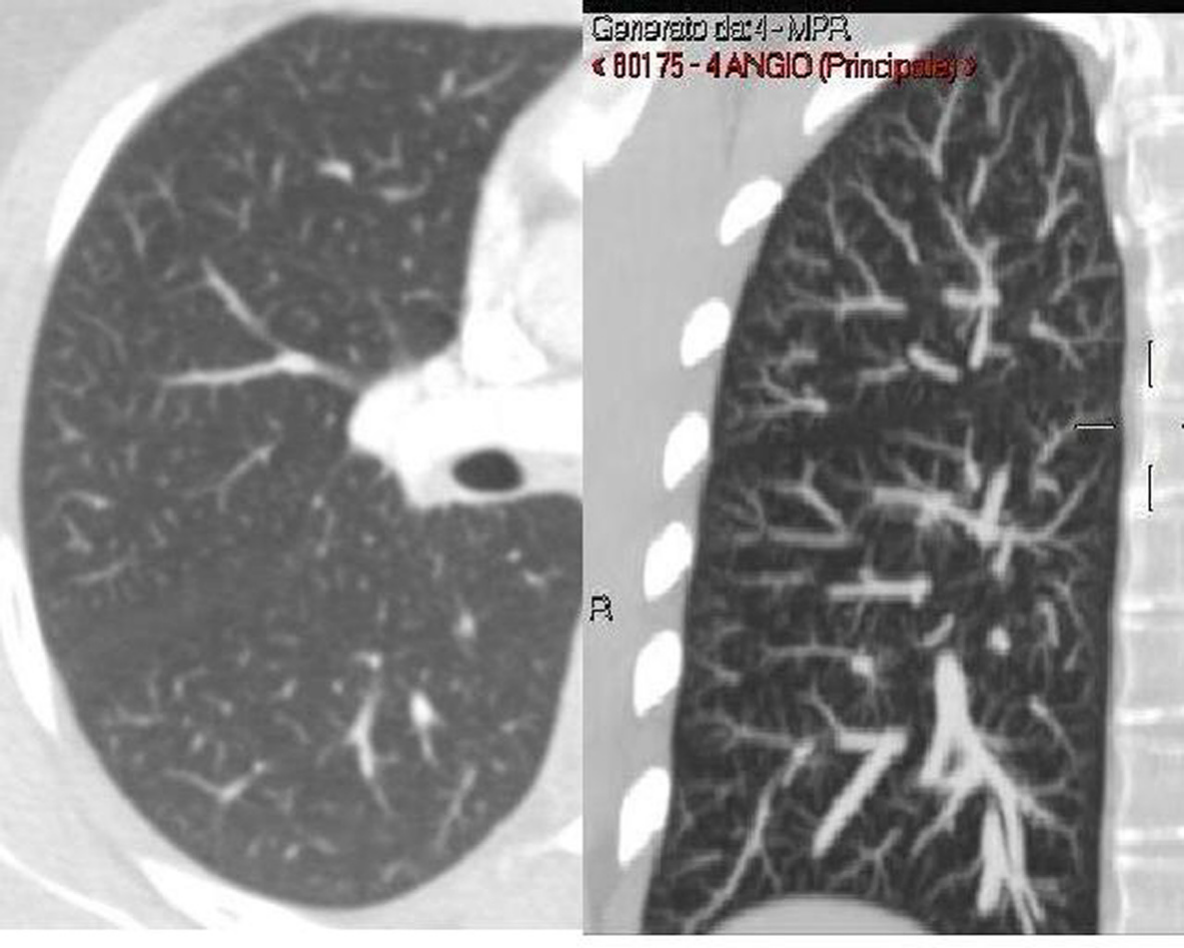
Sliding thin-slab MIP image obtained from single section CT angiographic data (section collimation 64x0.6 mm; slice thickness 2 mm; slice increment 1 mm; rotation time 0.5 sec) reveals centrilobular vessel–associated micronodules connected by arcade-like dilated subpleural vascular branches.

The chest X-ray was normal and a CT scan, though it did not reveal interstitial involvement or thromboembolic disease of the lungs, was suggestive of the pathologic thickening of the peripheral vascular bed, (Figure1) revealing the presence of centrilobular micronodules connected by dilated subpleural vascular branches.

A TTE and TEE were performed without evidence of intracardiac defects, shunts, or of pulmonary hypertension. Moreover, a magnetic resonance imaging (MRI) of the chest was inconclusive for the detection of intracardiac or intrapulmonary shunts.

**Figure 2:**
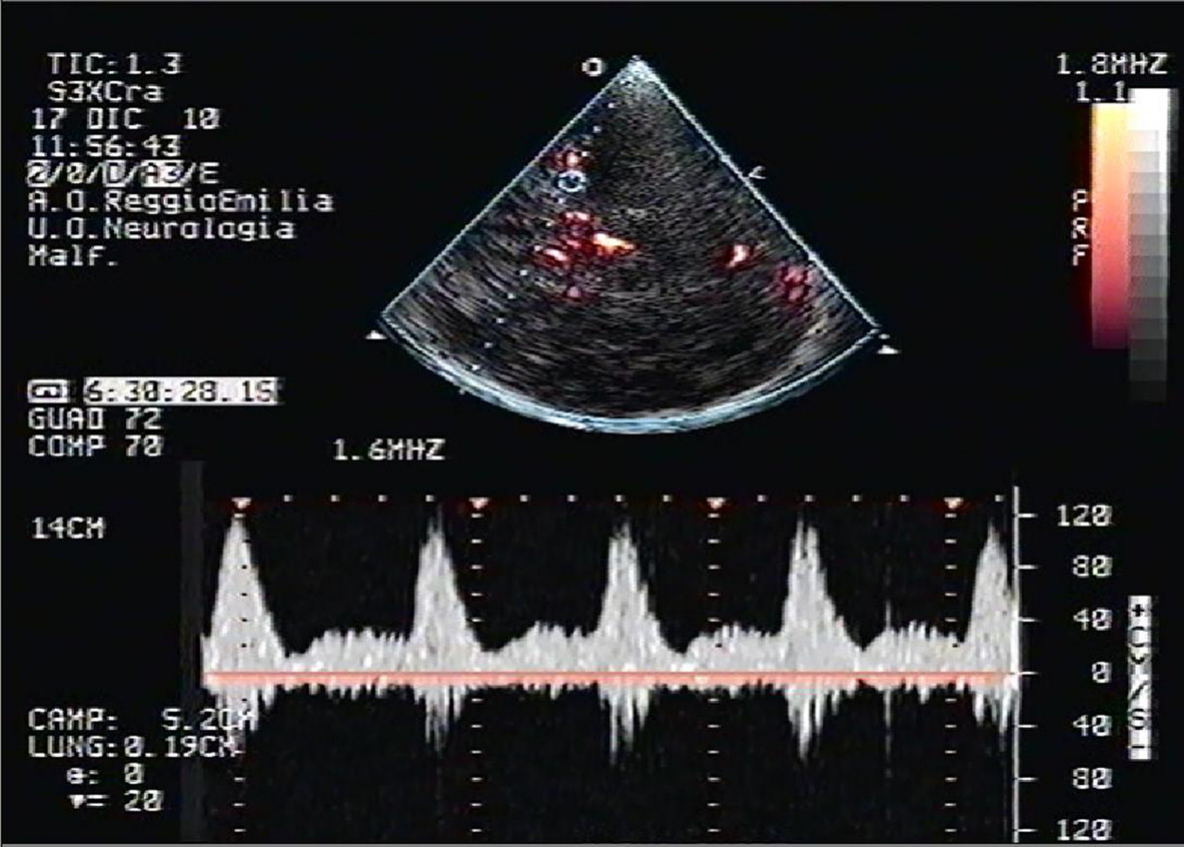
**Transcranial Colour-Coded Duplex Sonography (TCCS) from the temporal bone window in axial scanning plane with Power-mode.** The sampled right middle cerebral artery shows a continuous presence of multiple high-intensity transient signals (HITS), like a "curtain effect", 30 seconds after the release of the straining phase of the Valsalva manouver.

TCCS with saline contrast medium injection was performed according to Shariat and Coll.’s technique [8]. A mixture of saline solution (9 ml) and air (1 ml), agitated with 0.5 ml of the patient’s blood between two 10-ml syringes that were connected by a three-way stopcock, was injected into the right antecubital vein as a bolus and a Valsalva maneuver was elicited from the patient. The simultaneous monitoring of the right middle cerebral artery (MCA) showed the presence of multiple high-intensity transient signals (HITS) both during normal breathing and during the straining and relaxation phase of the Valsalva maneuver, confirming the presence of a continuous right-to-left shunt (Figure2). 

Further hepatologic evaluations emphasized the role of the chronic autoimmune cholangitis in an advanced stage; a hepatopulmonary syndrome was diagnosed and the patient was referred to a transplant centre and placed on a waiting list for liver transplant.

## Conclusions

In patients with a chronic liver disease, the presence of small artero-venous intrapulmonary RLS, can be responsible for a marked hypoxemia. The respiratory insufficiency developing from hepatopulmonary syndrome is usually difficult to diagnose by respiratory physicians as it develops in the absence of an intrinsic lung or heart disease.

This case report shows that right-to-left pulmonary shunts from hepatopulmonary syndrome can be suspected by standard CT imaging and MIP reconstruction of the pulmonary vascular bed and confirmed by a minimally invasive doppler transcranial examination with the use of agitated saline as contrast solution.

### Consent

Written informed consent was obtained from the patient for publication of this Case report and any and all accompanying images. A copy of the written consent is available for review by the Series Editor of this journal

## Abbreviations

(RLS): Right-to-left shunt; (TCCS): Transcranial Colour-Coded Duplex Sonography; (TTE): Transthoracic echocardiography; (TEE): Transesophageal echocardiography; (TCD): Transcranial Doppler; (MRI): Magnetic resonance imaging; (MCA): Middle cerebral artery; (HITS): Multiple high intensity transient signals.

## Competing interests

The authors declare that he/they have no competing interests.

## Authors’ contributions

CC conceived of the study, participated in its design and coordination and drafted the manuscript, MZ participated in the design of the study and performed the Transcranial Colour-Coded Duplex Sonography analysis, AC participated in the design of the study and drafting of the manuscript, NF participated in the design of the study and drafting of the manuscript, FM participated in the design of the study and drafting of the manuscript, LS participated in the design of the study and performed the MIP CT angiographic study, LZ participated in study design and coordination, drafting of the manuscript. All authors read and approved the final manuscript.

## Pre-publication history

The pre-publication history for this paper can be accessed here:

http://www.biomedcentral.com/1471-2466/12/42/prepub
